# Isolation, Characterization and Chemical Synthesis of Large Spectrum Antimicrobial Cyclic Dipeptide (l-leu-l-pro) from *Streptomyces misionensis* V16R3Y1 Bacteria Extracts. A Novel ^1^H NMR Metabolomic Approach

**DOI:** 10.3390/antibiotics9050270

**Published:** 2020-05-21

**Authors:** Ilhem Saadouli, Imène Zendah El Euch, Emna Trabelsi, Amor Mosbah, Alaeddine Redissi, Raoudha Ferjani, Imene Fhoula, Ameur Cherif, Jean-Marc Sabatier, Norbert Sewald, Hadda-Imene Ouzari

**Affiliations:** 1 LR03ES03, Laboratoire de Microorganismes et Biomolécules Actives, Faculté des Sciences de Tunis, Université Tunis Manar, 2092 Tunis, Tunisia; ilhem.saadouli@hotmail.fr (I.S.); imene.zendah@gmail.com (I.Z.E.E.); ferjaniraoudha@hotmail.fr (R.F.); fhoulaimene@gmail.com (I.F.); 2 Organic and Bioorganic Chemistry, Department of Chemistry, Bielefeld University, Universitätsstraße 25, 33615 Bielefeld, Germany; norbert.sewald@uni-bielefeld.de; 3 Higher Institute for Biotechnology (ISBST), LR Biotechnology and Bio-Geo Resources Valorization, University of Manouba, BVBGR-LR11ES31, BiotechpoleSidiThabet, 2020 Ariana, Tunisia; emna.trabelsi94@gmail.com (E.T.); amor.mosbah@isbst.uma.tn (A.M.); redissialadin@gmail.com (A.R.); ameur.cherif@uma.tn (A.C.); 4 Institute of Neuro Physiopathology (INP), Université d’Aix-Marseille, UMR 7051, Faculté de Pharmacie, 27 Bd Jean Moulin, 13385 Marseille CEDEX, France

**Keywords:** *Streptomyces misionensis*, antimicrobial activity, cyclic dipeptide (l-Leucyl-l-Proline)

## Abstract

*Streptomyces* is the most frequently described genus of *Actinomycetes*, a producer of biologically active secondary metabolites. Indeed, the *Streptomyces* species produces about 70% of antibiotics and 60% of antifungal molecules used in agriculture. Our study was carried out with the goal of isolating and identifying antimicrobial secondary metabolites from *Streptomyces misionensis* V16R3Y1 isolated from the date palm rhizosphere (southern Tunisia). This strain presented a broad range of antifungal activity against *Fusarium oxysporum*, *Aspergillus flavus*, *Penicillium expansum*, *Aspergillus niger*, *Candida albicans*, *Candida metapsilosis*, and *Candida parapsilosis* and antibacterial activity against human pathogenic bacteria, including *Escherichia fergusonii*, *Staphylococcus aureus*, *Salmonella enterica*, *Enterococcus faecalis*, *Bacillus cereus* and *Pseudomonas aeruginosa*. The purification procedure entailed ethyl acetate extract, silica gel column, and thin layer chromatography. Based on ^1^H NMR metabolomic procedure application, also supported by the GC-MS analysis, cyclic dipeptide (l-Leucyl-l-Proline) was identified as the major compound in the bioactive fraction. In order to confirm the identity of the active compound and to have a large quantity thereof, a chemical synthesis of the cyclic dipeptide was performed. The synthetic compound was obtained with a very good yield (50%) and presented almost the same effect compared to the extracted fraction. This study indicates for the first time that *Streptomyces misionensis* V16R3Y1 exhibits a broad spectrum of antimicrobial activities, produced cyclic dipeptide (l-Leucyl-l-Proline) and might have potential use as a natural agent for pharmaceutical and agri-food applications.

## 1. Introduction

The infectious diseases caused by multidrug-resistant microbes (MRS) are included in the list of the ten leading causes of death and productivity loss [1].Pharmaceutical and agrochemical industries produce many synthetic antibiotics and chemical fungicides, herbicides, and pesticides. However, the use of these chemicals must be strictly controlled because of their serious deleterious effects, including the high toxicity of chemotherapeutical drugs for humans and livestock, the development of resistance by pathogens, ecosystem disruption, and the decrease in the diversity of non-target organisms and environmental pollution [2]. Therefore, the natural sources of bioactive compounds has always sparked great interest from scientists as they provide a safe and effective alternative approach, which has high potential to control emerging MRS with minor impact on the environment [3].

*Actinomycetes* represent a treasure trove of new and potential drug molecules [4]. About 10,100 (45%) of the 22,500 analyzed bioactive secondary metabolites are from *Actinomycetes*. In particular *Streptomyces*, which is the principal genus of *Actinobacteria*, produce more than 7000 biologically active substances [5].

This fact has made *Streptomyces* the most used bacteria in many biotechnological fields including: medicine, veterinary practice, agriculture and the pharmaceutical industry [4]. *Streptomyces* is characterized by a complex morphologic differentiation cycle accompanied by the synthesis of an infinite variety of secondary metabolites that belong to various classes such as glycopeptides, polyketides, aminoglycosides, tetracycline, β-lactams, nucleosides, terpenoids, alkaloids, macrolides, and others [6].

Cyclic dipeptides (also known as 2,5-diketopiperazines, DKPs, 2,5-dioxopiperazines, DOPs) are a class of small molecules that have been reported to be produced as extracellular secondary metabolites by *actinomycetes* [7].

Since the beginning of the 20th century, the interest in those chiral molecules has significantly increased due to their properties and several characteristics that make them attractive scaffolds for drug discovery. Therefore, their core structure appears in several drugs such as amphomycin, bicyclomycin, brevianamide F, verruculogen and spirotryprostatin B [8]. They are small and conformationally constrained scaffolds in which functional groups can be introduced at up to six positions and stereochemistry controlled at up to four positions. Cyclic dipeptides also have high stability and resistance to protease and human digestion. They contain 2 *cis*-amide bonds and as a result possess 2 H-bond acceptors and 2 H-bond donors. Due to this chemical structure, they have the ability to bind to a wide range of receptors and enzymes. In addition, they have a rigid backbone due to their structural chirality, which can mimic a preferential peptide conformation being in either a planar or boat form. DKPs contain constrained amino acids embedded within their structures without the limitation properties of conventional peptides [9].

As a part of our research program for bioactive compounds from extremophilic *actinomycetes*, a terrestrial bacterium was isolated from the Sahara of the southern region of Tunisia (Tunisian oasis soil) and identified as *Streptomyces misionensis* strain V16R3Y1. This strain has presented a potent antifungal effect against *Fusarium oxysporum*, *Aspergillus flavus*, *Penicillium expansum*, *Aspergillus niger, Candida albicans*, *Candida metapsilosis*, and *Candida parapsilosis* and antibacterial activity against human pathogens, including *Staphylococcus aureus, Escherichia fergusonii, Salmonella enterica, Enterococcus faecalis, Bacillus cereus* and *Pseudomonas aeruginosa*.

Secondary metabolites are generally extra cellular. In fact, their isolation in highest purity from the culture medium requires the use of different solvents and the application of various chromatographic techniques and analytical systems such as thin-layer chromatography (TLC), gas chromatography-mass spectrometry (GC-MS) and nuclear magnetic resonance (NMR). Therefore, we proceed to up-scale the strain growth at optimum conditions, followed by different solvent extractions, and chromatographic separation of the fermented broth of *Streptomyces misionensis* strain V16R3Y1 afforded one active fraction. Further, for the structural elucidation of bioactive metabolites, a metabolomic procedure using ^1^H NMR analysis (usually used in the identification of metabolic compounds in serum or in urine) was applied initially in the identification of the major compound in the bioactive fraction, thus allowing the confirmation of the results obtained using GC-MS.

These results indicated for the first time that the species of *Streptomyces misionensis* was able to produce antibiotics related to cyclic peptides and might have potential use as a natural antibacterial and antifungal agent for pharmaceutical and agricultural applications. In order to confirm the potential application of this cyclic dipeptide, we adapted an ancient Boc/Benzyl protocol used in the chemical synthesis of the cyclic dipeptide l-Leucyl-l-Proline, which gave a very good yield (50%). The activity of the synthetic cyclic dipeptide presented almost the same effect of the biological fraction.

## 2. Materials and Methods

### 2.1. Chemicals and Media

All the reagents and chemicals used for extraction, column chromatography (CC), thin layer chromatography (TLC), gas chromatography–mass spectrometry (GC–MS), NMR and chemical synthesis were HPLC grade (such as Fmoc-Pro-OH, Fmoc-Leu-OH, HOBt, EDC, DMF, DIPEA, DCM, piperidine, LiOH and MeOH), and were purchased from sigma Aldrich and Millipore. Column chromatography was carried out on silica gel 60 (0.040–0.063 mm, Merck, Darmstadt, Germany). TLC was performed with pre-coated Merck silica gel 60 _PF254+366_ (Merck, Darmstadt, Germany). Tryptic soy Broth (TSB) was purchased from Biolife.

### 2.2. Microbial Strains Origin

*Streptomyces misionensis* V16R3Y1 strain was isolated from Tunisian oasis soil and found to be a potent antimicrobial activity producer. The procedures used for microbial isolation, maintenance, and identification as well as its GenBank accession number (KJ956655.1) were reported previously by Ferjani et al. [10].

Antimicrobial activities were tested against a broad range of micro-organisms. The test pathogens used in this study were as follows: Gram-positive bacteria such as *Staphylococcus aureus, Bacillus cereus* NR074540.1, *Enterococcus faecalis* MK584170; Gram negative bacteria such as *Escherichia fergusonii* MK584171, *Pseudomonas aeruginosa* MK584172, and *Salmonella enterica* MK584173; medically important dermatophytes such as *Candida albicans* MK599152, *Candida metapsilosis*: MK599150, *Candida parapsilosis* MK599151 and agriculturally important fungi such as *Fusarium oxysporum*, *Penicillium expansum*, *Aspergillus flavus* and *Aspergillus niger*. All strains were obtained from the microbial collections of the Laboratory of Microorganisms and Active Biomolecules (LMBA), Faculty of Sciences of Tunis.

### 2.3. Antimicrobial Activity Tests

Antimicrobial activity of crude extract and the eluted fractions was determined by the agar well diffusion method according to the National Committee for Clinical Laboratory Standards (NCCLS) [11] with slight modifications. Wells of diameter 8 mm were punched into the agar medium and filled with 10 μL of corresponding sample and allowed to diffuse at room temperature for 2 h. Plates were covered by 3 mL of top agar containing 50 μL of culture of test strains. Subsequently, the plates were then incubated in the upright position at 37 °C for 24 h for bacteria and at 28 °C for 48 h for fungi. Wells containing the same volume of methanol/dichloromethane (MeOH/DCM) served as negative controls. After incubation, the diameters of the growth inhibition zones were measured in mm. Three replicates were carried out for each sample against each of the test organisms.

### 2.4. Minimum Inhibitory Concentration (MIC) Assay

The antimicrobial spectrum of the synthetic *cyclo*-(l-pro-l-leu) (SP) was determined in terms of minimal inhibitory concentration (MIC) against gram-positive and gram-negative bacteria and fungi using the agar plate diffusion assay [12].

### 2.5. Screening of Solvent for the Extraction of Antimicrobial Compound V16R3Y1

**Figure 1:**
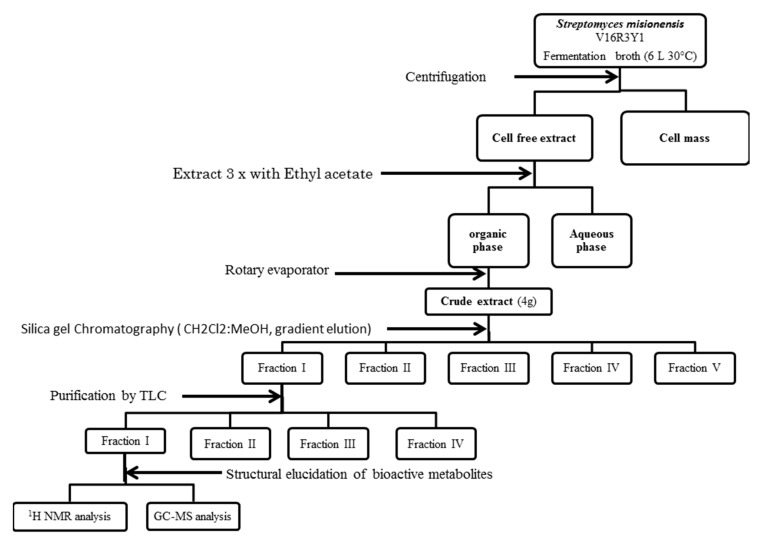
Work-up schema for the purification of the active molecules of the ***Streptomyces misionensis*** V16R3Y1.

The protocol used in the extraction of antimicrobial compounds was divided into two steps: Firstly, the strain *Streptomyces misionensis* was cultivated in a 3 flask of 250 mL comprising 100 mL of production TSB broth medium under shaking at 28 ± 2 °C and 150 rpm for 10 days. After incubation, the culture supernatant was obtained by centrifugation at 10,000 rpm at 4 °C for 15 min and subjected to a freeze-dry process. The solid dry product was extracted 3 times with equal volumes of three different solvents (ethyl acetate, acetone, and methanol) separately. The organic phases were concentrated under vacuum using a rotary evaporator at 40 °C to obtain the crude extract. Each dried crude extract was tested for antimicrobial and antifungal activities using the above described well diffusion agar method. It has been shown that the most adequate solvent is the ethyl acetate. We decided to continue working with this solvent. Secondly, the strain *Streptomyces misionensis* was cultivated in a 6 flask of 2.5 L comprising 1 L of production TSB broth medium under shaking at 28 ± 2 °C and 150 rpm for 10 days. After incubation, the culture supernatant was obtained by centrifugation at 10,000 rpm at 4 °C for 15 min. The supernatant was extracted 3 times with equal volumes of ethyl acetate. The aqueous phase was discarded, and the organic phase was passed through a pad of anhydrous sodium sulphate to remove excess water and thereafter evaporated to dryness using a rotary vacuum evaporator at 40 °C to obtain the crude extract. The whole procedure is described in Figure 1.

### 2.6. Purification of Bioactive Compounds

The ethyl acetate extract obtained after drying was then loaded on a silica gel column (60 × 3 cm) previously equilibrated with 100% dichloromethane and eluted successively by 0.5 L DCM -MeOH [95:5, *v*/*v*], 0.5 L DCM -MeOH [90:10 *v*/*v*], 0.5 L DCM -MeOH [80:20, *v*/*v*], 0.5 L DCM -MeOH [70:30 *v*/*v*], 0.5 L DCM -MeOH [60:40, *v*/*v*] and 0.5 L DCM -MeOH [40:60, *v*/*v*]. Five fractions were obtained by FI–V. Each pooled fraction was concentrated to dryness with a rotary evaporator and was checked for its potentiality against the test pathogens considered in the current study. Inhibitory activities were found in the fraction eluted by MeOH/(DCM) (5:95, *v*/*v*). Almost no inhibition growth on pathogenic test strains was observed for the other isolated fractions.

### 2.7. Thin Layer Chromatography (TLC) Procedure

The active fraction was applied to a silica gel TLC plate using the capillary tube. A row of spots of the active fraction was applied 1.5 cm above the bottom of the TLC plates. The spots were left to dry. The TLC plate was placed vertically in a developing beaker containing MeOH/DCM (5:95, *v*/*v*) as a solvent system and covered with the glass in order to prevent the evaporation of the solvents. Chromatography was allowed to run until it moved up to 80% of the TLC plate. After visualization with UV light under 365 nm and 254 nm, the component having the same retention factor was scraped from the silica gel. The substances present in the silica gel were extracted with 4-fold amounts of MeOH/DCM (5:95, *v*/*v*). Each extract was concentrated to dryness, dissolved in a small amount of solvent and then was checked for bioactivity using the same method of the well diffusion agar. The chemical composition of the bioactive fraction (BF) was investigated using gas chromatography and ^1^H-NMR.

### 2.8. Gas Chromatography–Mass Spectrometry (GC–MS) Analysis

The samples were solubilized in methanol and analyzed using an Agilent GC 7890BMS 240 ion trap gas chromatography (GC) system, equipped with MS detector and HP-5MS capillary column (30 m × 250 µm, film thickness 0.25 µm). Injector temperature was set at 280 °C and GC oven temperature was started at 40 °C for 2 min, then increased by 5 °C/min to 250 °C and held 20 min at this temperature. Analysis was carried in full scan mode for 60 min. The gas carrier helium was used at a flow rate of 1 mL/min. Samples of 1 µL were injected with the split mode and an ionization range from 50 to 1000 mV. Identification of metabolites was done by comparing their mass spectra with those referenced in the Library (NIST).

### 2.9. ^1^H Nuclear Magnetic Resonance (NMR)

The dried TLC extract fractions were dissolved in chloroform-d. Tetramethylsilyl was used as an internal standard. Measurements were carried out at 25 °C using a Bruker AV-500 NMR instrument operating at a proton NMR frequency of 500.13 MHz. For each sample, 96 scans were recorded using the same parameters as previously reported by El Euch et al. [13]. Spectral intensities were scaled to the total intensity for the chloroform extract and reduced to the integrated regions of equal width (0.04 ppm) corresponding to the region *δ_H_*1–12 ppm. The region of *δ_H_* 7.18–7.3 ppm was excluded from the analyses because of the residual signal of chloroform.

### 2.10. Application of Metabolomic Procedure for the Identification of the Extracted Metabolites

The general procedure of the identification of the metabolites in the biological fraction was carried out using a new protocol adapted from the metabolomic protocol identification. The processing was assessed by the routine Chenomx Processor (software routine for metabolomic phase correction, baseline correction, and chemical shift referencing) [14,15,16,17].

The spectra were then transferred to the Chenomx Profiler (another module in Chenomx), followed by annotation of all the peaks using topspin and Chenomx Discovery. Chemical shift of proton metabolites were compared to the published NMR spectra of the synthetic product [18] and to the internal database (distributed with the Chenomx software).

### 2.11. Synthesis of the Cyclo Dipeptide (Leu-Pro) in Solution 

The method used in the synthesis of cyclo-(l-pro-l-leu) was derived from the method described by Campbell [19]. In their protocol the author used a methylated proline and a boc strategy; in our case we use a methylated leucine and a fmoc strategy. The general protocol is described as follows:

**Coupling reaction** The Fmoc protected l-*α*-amino acid (1 mmol), the l-*α*-aminoacid ethyl ester (1 mmol), HOBt (1 mmol) and EDC (1 mmol) were dissolved in DMF (10 mL). The pH was adjusted to 8 by addition of DIPEA (700 μL).The solution was left at room temperature for 24 h. The solution was concentrated *in vacuo* and DCM (50 mL) was added. The obtained residue was dried under high vacuum and used for further reactions.

**Fmoc-cleavage** The Fmoc-protected compound (1 mmol) was dissolved in 20% piperidine in DMF (10 mL) and left at room temperature for 20 min. The reaction solution was concentrated *in vacuo* at 40 °C. The residue was dried under high vacuum and used for further reactions.

**Ester saponification** The ester (1 mmol) was dissolved in DMF/H_2_O (9:1, 4 mL) and 3M LiOH in water (1.3 mL) was added. The solution was kept at 50 °C for 16 h. The reaction solution was concentrated *in vacuo*. The residue was dried under high vacuum, and then used for cyclization.

**Cyclization** The linear dipeptide (1 mmol) was dissolved in DCM (100 mL). This solution was combined with a solution of EDC (1.5 mmol) and HOBt (1.5 mmol) in DCM (234 mL). DIPEA (3 mmol) was added to adjust the pH to 8. The reaction solution was kept at room temperature for 24 h.The solvent was concentrated *in vacuo* and 50 mL of DCM was added. The residue was then chromatographed on silica gel DCM/MeOH (90:10, *v*/*v*), allowing a yield of 50%.

### 2.12. Liquid Chromatography–Mass Spectrometry (HPLC–MS) Analysis

Experiments were performed on LC/MS (LC-2010A HT Liquid Chromatograph Shimadzu, Marne la Vallée, France) using an ESI source operated under negative mode. LC/MS separation was achieved on a C18 Phenomenex 150 mm 4.6 mm at 35 °C. The detection wave lengths were 214 and 280 nm. Elution was carried out with solvents A and B. A linear gradient from 0% to 60% solvent B for 60 min was used. The flow rate was set up at 1 mL/min. The mass of the product has been calculated according to the following are: *m*/*z*+H+ as formula: *m*/*z* = (Mproduct + (zM*adduct))/z. Adducts products are: *M*/*z*+H+ (1 g/mol), or *M*/*z*+Na+ (23 g/mol), or *M*/*z*+K+ (39.1 g/mol).

## 3. Results

### 3.1. Extraction and purification of Bioactive Compounds

Three different polar and non-polar solvents (ethyl acetate, methanol, and acetone) were used. Only the ethyl acetate extract had an effective bioactivity against the tested pathogens, whereas acetone and methanol extracts did not show any activity. On the basis of chromatography column, five fractions were obtained. Activities of the five fractions were tested. The MeOH/DCM (5:95, *v*/*v*) fraction presented the highest antimicrobial potential. Hence, it was again purified in TLC chromatography to produce 4 fractions. The biological fraction which presented an antimicrobial activity against all the tested pathogens was investigated by GC-MS and ^1^H-NMR.

### 3.2. Gas Chromatography–Mass Spectrometry (GC–MS) Analysis

The TLC-derived active fraction, which appeared as light brown, was soluble in chloroform, dichloromethane, dimethyl sulfoxide, and isopropanol. The compounds of the isolated fraction were identified using GC-MS analyses. The spectra were analyzed from the available library data NIST MS. The identified compounds with their retention times are listed in Table 1. The GC-MS analysis revealed the presence of 5 compounds. The most abundant compound detected in the bioactive fraction was hexahydro-3-(2-methylpropyl) pyrrolo [1,2-a] pyrazine-1, 4-dione followed by *N*-valeryl-l-proline decyl ester and the minor compounds were benzene acetamide, 2-(ethylhexyl)-hexylsulfate, and 5-isopropylidene-3,3-dimethyl-dihydrofuran-2-one.

### 3.3. NMR Spectra Analysis with the New Procedure

**Figure 2:**
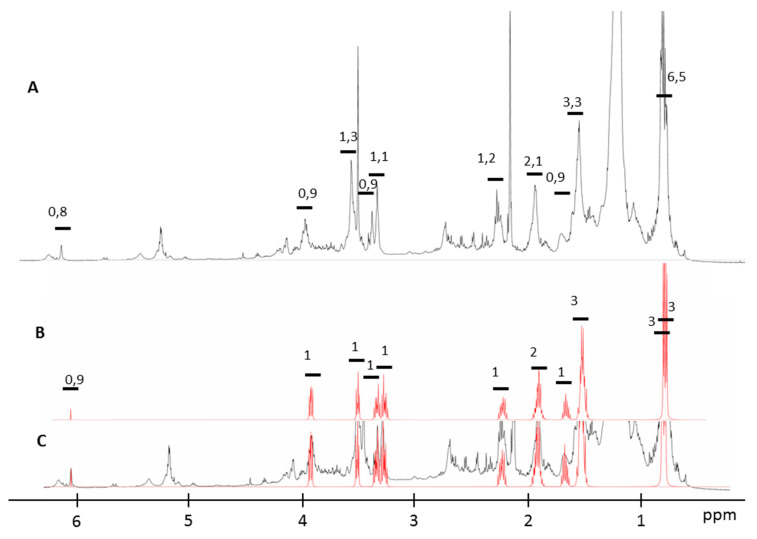
(**A**) ^1^H NMR spectrum of the biological active fraction, (**B**) ^1^H NMR spectrum of The synthetic cyclo-(l-pro-l-leu) and (**C**) superposition of (**A**) identified cyclo-(l-pro-l-leu) in the biological fraction with the NMR spectra of (**B**) the synthetic cyclo-(l-pro-l-leu).

The NMR metabolomics procedure was adapted in this work for the qualitative and quantitative determination of the metabolites present in the active fraction. NMR spectra analysis of the biological fraction using the Chenomx software revealed the presence of hexahydro-3-(2-methylpropyl) pyrrolo [1,2-a]pyrazine-1,4-dione as the major product. The identified DKP cyclic dipeptide was composed of two amino acids in the L configuration (l-leu and l-pro). This new procedure allows us, as well, to identify the stereoisomerism form of this compound. The superposition of the NMR spectra of the synthetic *cyclo*-(l-pro-l-leu) and that of the biological fraction is presented in Figure 2.

### 3.4. Cyclo Dipeptide Synthesis

*Cyclo*-(l-pro-l-leu) was synthesized according to the procedure for the solution strategy. l-Leucine-OCH_3_ (20 mmol, 2.90 g) was obtained by the reaction between the l-Leucine (25 mmol, 3.25 g) and SOCl_2_ (30 mmol, 3.24 g) in methanol (30 mL). ESI-MS: [M+H]^+^*m*/*z* = 146.17; Fmoc-l-Pro-OH (6.74 g, 20 mmol), l-Leu-OCH_3_ (2.90 g, 20 mmol), EDC (3.83 g, 20 mmol), HOBt (2.70 g, 20 mmol), *N*,*N*-dimethyl formamide (DMF, 40 mL), disopropyl ethylamine(DIEPA) to pH = 8. Yield: 9 g, 18.6 mmol, 94%. ESI-MS: [M+H]^+^*m*/*z* = 465.54 Da. 18.6 mmol (9 g) of the Fmoc-protected compound was reacted. Yield: 4.35 g, 16.7 mmol, 90%. ESI-MS: [M+H]^+^*m*/*z* = 243.3. 16.7 mmol (4.35 g) of the ester was reacted. Yield: 3.2 g, 13.4 mmol, 80%. ESI-MS: [M+H]^+^*m*/*z* = 229.3. 13.4 mmol (3.2 g) of dipeptide was reacted. Crude product: 1.6 g, 6.5 mmol, 50%. The crude product was purified by chromatography on silica gel DCM/MeOH (90:10, *v*/*v*). ESI-MS: [M+H]^+^*m*/*z* = 211.3 This result is in accordance with the result obtained by Campbell [19].

### 3.5. Biological Activity of Biological Fractionand Synthetic Peptide

**Figure 3:**
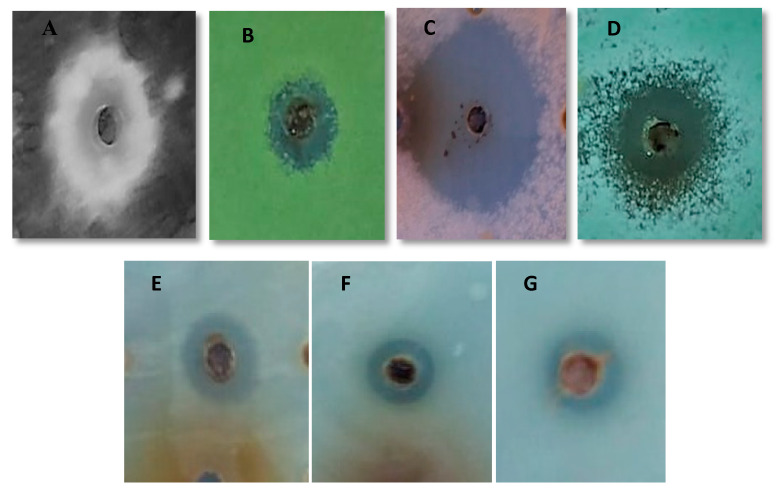
Inhibition Zone of active fraction against (**A**) Aspergillus niger, (**B**) Aspergillus flavus, (**C**) Fusarium oxysporum, (**D**) Penicillium expansum, (**E**) Candida albicans, (**F**) Escherichia fergusonii and (**G**) Enterococcus faecalis.

Antimicrobial activity in terms of inhibition zone of biological fraction and the synthetic peptide and the MIC of synthetic peptide against different microorganisms including bacteria, dermatophytes and filamentous fungi are indicated in Table 2 and Figure 3.

As shown in Table 2 both the biological fraction and the synthetic peptide Cyclo (l-leu-l-pro) possess antimicrobial activity against all the tested pathogenic organisms. Whereas the synthetic peptide Cyclo (l-leu-l-pro) displayed superior activity with inhibition zone range from 6 mm to 40 mm diameter while for biological fraction these values ranged from 5 mm–37 mm. This might be due to the high degree of purity of synthetic peptide compared to the partially purified peptide obtained from the strain *Streptomyces misionensis*.

The antimicrobial activity of the cyclo (l-leu-l-pro) was also investigated by MICs; we found that MICs for all the organisms varied from 230 to 11 μg/mL. Significant susceptibility against cyclo(leu-pro) was observed in the tested fungi, namely *Fusarium oxysporum*, *Aspergillus flavus*, *Aspergillus niger*, and *Penicillium expansum*, which exhibited MIC values of 16, 16, 17, and 18 μg/mL respectively. The MIC values for cyclo (l-leu-l-pro) against *Candida albicans*, *Candida metapsilosis* and *Candida parapsilosis* were 50 μg/mL, 32 μg/mL, and 30 μg/mL, respectively. Among the gram-negative bacteria tested, *Escherichia fergusonii* showed the higher level of resistance toward cyclo (l-leu-l-pro) with an MIC value of 230 μg/mL and *Salmonella enterica* showed the lower level with an MIC value of 11 μg/mL. Among the gram-positive bacteria, *Enterococcus faecalis* was found to be the most sensitive to the cyclo dipeptide followed by *Bacillus cereus* and *Staphylococcus aureus*.

## 4. Discussion

It is a widely known fact that the increasing levels of antibiotic resistance among pathogenic bacteria and fungi in agriculture and humans have a critical role in various diseases. This leads to the investigation of potential bioactive compounds from natural sources. A wide spectrum of sources is available for natural antimicrobials, among which *actinomycetes* occupy a key position. They are well-known as a prominent source for finding novel biologically active secondary metabolites such as antibacterial, antifungal, antitumor, immunosuppressive and antioxidant compounds.

Among a collection of bacteria isolated from the rhizosphere of date palm from southern Tunisia, the isolate *Streptomyces misionensis* V16R3Y1 has an interesting bioactivity profile. Hence, the isolate was selected for further investigations: large-scale cultivation, extraction, isolation, purification and identification of its different antimicrobial compounds.

The selection of the appropriate solvent is a crucial step for the isolation of bioactive molecules. In the current study, among the three different solvents used to extract the antimicrobial compounds, the ethyl acetate extract showed the maximum activity against all the pathogens tested. It has already been reported by Thirumurugan et al. [20] that the ethyl acetate extract showed maximum antibacterial efficacy on all tested pathogens. Baskaran et al. [21] has also reported that out of five different solvents used to extract the antimicrobial compounds of *Streptomyces parvulus*, the ethyl acetate extract showed remarkable activity against the eleven pathogenic bacteria and the six tested fungal pathogens.

Metabolomic procedures have recently been described as a new method for the identification of metabolites from mixtures. We describe here for the first time the elucidation of the structure of the five compounds, as well as their corresponding quantity. Furthermore, metabolomic analysis of NMR spectra allowed us to determine the stereoisomerism of the major compound present in the active fraction. NMR spectra of pattern recognition methods in metabolomics has enabled the identification and quantification of metabolites [22] in different biological samples including biofluids such as urine [23], serum [24,25] and synovial fluid [26] and in cell pellets [27], thus providing highly informative data about the functional state and the composition of living organisms.

Based on the identified chemical constituents using GC-MS and NMR analysis, the bioactive purified fraction was mostly composed of diketopiperazines. We focused our study on this cyclic dipeptide. DKPs derived from the secondary metabolism of microbes constitute an interesting group of molecules for the development of new therapeutic agents. However, the difficulty with natural products is that they require tedious and time consuming purification to identify these compounds from complex mixtures, often in tiny amounts. Therefore, methods of chemical synthesis form an effective and a tractable alternative to the production of these molecules in high quantities. In our study we have performed a chemical synthesis of this cyclic peptide in solution to obtain a sufficient quantity of the dipeptide and confirm the activity origin. We applied a fmoc/trt strategy to synthesize our product. This method is inspired by the Boc/benzyl strategy used by *Campbell* [19]; the main difference is in using an O-methylated Leucine instead of an O-methylated proline and a fmoc-proline instead of Boc-leucine. We can propose this strategy as an efficient solution-phase synthetic route to cyclic dipeptide (leu-pro). In brief, L-Leu-OMe was coupled to N-Fmoc-protected amino acids using standard EDC and HOBt-mediated conditions. After cleavage of the N-Fmoc group under basic conditions (20% piperidine), intramolecular cyclization proceeded smoothly at room temperature upon treatment with EDC, HOBt and DiPEA to generate DKPs in sufficient yields for biological evaluation (50% yield; 800 mg scale). This method is considered less toxic than the Boc strategy which uses a toxic solvent HF. The yield of our synthesis strategy is more efficient than that of the strategy described by Campabell, 50% and 38% respectively. We have noticed that the activity of the synthetic product present almost the same activity of the bioactive fraction. We can postulate that the dipeptide *cyclo*-(l-leu-l-pro) was responsible for the antimicrobial activity of this strain. The result obtained for the MIC of synthetic peptide against *B. cereus*, *E. coli*, *S. aureus* was comparable with that obtained for *cyclo*-(l-leu-l-pro) previously isolated from *Bacillus* sp [28]. A study by Mangamuri [29] has demonstrated that Cyclo (D-pro-D-leu) form possesses a MIC of 256 μg/mL and 64 µg/mL against *F. oxysporum* and *S. aureus*, respectively, whereas the synthetic peptide Cyclo (l-pro-l-leu) displayed superior activity with 16 and 30 μg/mL against *F. oxysporum* and *S. aureus* respectively. This can be explained by the chirality variation in the amino acids, which plays a crucial role in biochemical systems and enantiomers often have various physiological behaviors [30].

Among the many cyclo-dipeptides isolated from nature, cyclo-(l-leu-l-pro) has been isolated from many living organisms, including bacteria such as *Rosellinia necatrix* [31] and *Lactobacillus plantarum* [32] and fungi such as *Aspergillus ochraceus* [33] and *Cordyceps sinensis* [34]. Furthermore, recent findings conducted on this compound isolated from *Bacillus* Sp. demonstrated high antimicrobial activity against bacteria and fungi, especially against plant pathogenic fungi [28]. Moreover, another study demonstrated that the *cyclo*-(l-leu-l-pro) produced by *Achromobacter xylosoxidans* inhibits aflatoxins production by *Aspergillus parasiticus* [35].

Besides its antimicrobial properties, *cyclo*-(l-leu-l-pro) is effective as a seed defense biopriming (SDB) agent, and can be used to induce plant resistance against *P. syringae* pv. lachrymans in cucumber and against *X. axonopodis* pv. vesicatoria in pepper [36].

*cyclo*-(l-leu-l-pro) was also detected in various *Streptomyces* species, for example *Streptomyces albus* CN-4 [37] and *Streptomyces omiyaensis* SCH [38]. The detection of heterocyclic organic compound belonging to the DKP group in the extract of the species *Streptomyces misionensis* is deemed as one of the most important findings in the current study. Thus, it was suggested that the identified diketopiperazines are the main compound responsible for the antimicrobial activities observed in *Streptomyces misionensis* extract.

## 5. Conclusions

In summary, this study demonstrates for the first time that *Streptomyces misionensis* V16R3Y1 exhibits a broad antimicrobial spectrum, as it inhibited Gram-positive and Gram-negative bacteria, as well as fungi. According to the GC-MS analysis and NMR Data, the major constituent of the biological fraction was the pyrrolo pyrazine and was suggested to be the major contributing factor for observed antibacterial and antifungal activities. The identification of diketopiperazines from *Streptomyces misionensis* species is reported here for the first time.

## Figures and Tables

**Table 1:** Compounds obtained from the active biological fraction produced by *Streptomyces misionensis* strain V16R3Y1 by GC-MS analysis.

Retention Time; (Peak Area %)	*m*/*z*	Name of the Compound	Chemical Structure	Activity ^a^
7.614 (10.088)	134.89; 91.88; 90.92; 64.61	Phenylacetamide	MF: C_8_H_9_NO MW: 135.166 g/mol 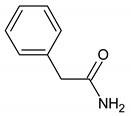	Drug, antimalarial
9.247 (2.880)	84.91; 70.88; 56.74; 54.73	Hexyl-(2-ethylhexyl)sulfate	MF: C_14_H_30_O_3_S MW: 278.451 g/mol 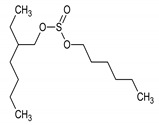	Non active
10.859 (28.241)	153.88; 69.59; 56.66	*N*-Valeryl-l-proline decyl ester	MF: C_20_H_37_NO_3_ MW: 339.5 g/mol 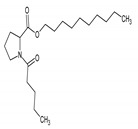	Non active
11.004 (1.758)	153.96; 54.57; 69.67	5-Isopropylidene-3,3-dimethyl-dihydrofuran-2-one	MF: C_9_H_14_O MW: 154.206 g/mol 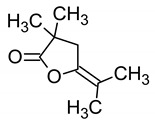	Non active
11.669 (57.033)	154.04; 124.91; 70.88; 69.59	*cyclo*-(l-prolyl-l-leucine); *cyclo*-(l-leu-l-pro)	MF: C_11_H_18_N_2_O_2_ MW: 210.272 g/mol 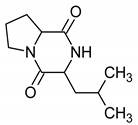	-Antimicrobial -Antitumoral -Antiviral -Cytotoxic and neuroprotective effects

^a^: PubChem: https://pubchem.ncbi.nlm.nih.gov/.

**Table 2:** Inhibition zone of the biological fraction (BF) and the synthetic peptide (SP), and the minimal inhibitory concentration (MIC) of (SP) on the tested microbes.

Test Microbes	Inhibition Zone (mm) BF (400 μg)	Inhibition Zone (mm) SP (300μg)	SP MIC (μg/mL) ± SD
*Fusarium oxysporum*	37	40	16 ± 1.00
*Aspergillus flavus*	13	15	16 ± 1.70
*Penicillium expansum*	24	27	18 ± 1.00
*Aspergillus niger*	13	15	17 ± 0.00
*Candida albicans*	10	12	50 ± 1.00
*Candida metapsilosis*	9	10	32 ± 1.00
*Candida parapsilosis*	8	9	30 ± 2.60
*Escherichia fergusonii*	5	6	230 ± 1.00
*Salmonella enterica*	7	8	11 ± 0.00
*Enterococcus faecalis*	6	7	12 ± 1.70
*Bacillus cereus*	5	7	16 ± 1.00
*Staphylococcus aureus*	5	6	30 ± 1.00
*Pseudomonas aeruginosa*	5	6	34 ± 1.70
